# *Ancylostoma ceylanicum* Hookworms in Dogs, Grenada, West Indies

**DOI:** 10.3201/eid2809.220634

**Published:** 2022-09

**Authors:** Patsy A. Zendejas-Heredia, Vito Colella, Maxine L. A. Macpherson, Wayne Sylvester, Robin B. Gasser, Calum N. L. Macpherson, Rebecca J. Traub

**Affiliations:** The University of Melbourne, Parkville, Victoria, Australia (P.A. Zendejas-Heredia, V. Colella, R.B. Gasser, R.J. Traub);; St. George’s University, St. George’s, Grenada (M.L.A. Macpherson, W. Sylvester, C.N.L. Macpherson); 1 These authors contributed equally to this article.

**Keywords:** *Ancylostoma ceylanicum*, zoonoses, hookworms, soil-transmitted helminths, epidemiology, dogs, parasites, Grenada, West Indies

## Abstract

*Ancylostoma ceylanicum* hookworms are recognized agents of human infection in the Asia–Pacific region. We investigated prevalence of zoonotic hookworm infections in dogs in Grenada in 2021; 40.8% were infected by hookworms, including *Ancylostoma ceylanicum*. Surveillance of this parasite in dogs and humans is needed in tropical/subtropical countries in the Americas.

Hookworms are blood-feeding enteric parasites that infect >700 million persons worldwide and cause detrimental health outcomes such as iron deficiency anemia, protein malnutrition, and impaired growth, particularly in children (*1*). Although most infections are attributed to anthroponotic species of hookworm, dogs serve as a reservoir for transmission of zoonotic species to humans (*1*,*2*). Of those hookworm species, only *Ancylostoma ceylanicum* can complete its life cycle in humans and dogs; both species of mammals contribute to environmental contamination with eggs (*1*).

Traditionally, the diagnosis of hookworm infection has relied on detecting egg in fecal samples (*3*). Because of the morphologic similarity of hookworm eggs, it is not possible to definitively differentiate these parasites at the species level by light microscopy alone (*1*,*3*). In the past decade, copromolecular tools, such as multiplex quantitative real-time PCR (M-qPCR), have enabled molecular differentiation of hookworm species in fecal samples from dogs and humans (*1*). These molecular tools are contributing to knowledge of the distribution of these parasites, particularly *A. ceylanicum*, which is now recognized as the second most common species infecting humans in the Asia–Pacific region (*2*). Despite early reports of *A. ceylanicum* hookworms in humans in Brazil, in dogs in Suriname in the early 1900s (*1*,*4*), and recently in a child living in Germany who most likely acquired the infection while in Colombia (*5*), endemicity of this parasite in dogs in the Americas has not been molecularly confirmed.

Grenada is a tropical small island developing state located in the West Indies and comprises 3 main islands (Grenada, Carriacou, and Petite Martinique) and many smaller uninhabited islands (*6*). Grenada has a population of ≈112,579 persons, 23.2% of whom are children <14 years of age (*6*) and of ≈35,000 dogs, a high proportion of which are pothounds (strays, mongrels) that roam freely; few receive veterinary care or anthelmintic treatment (*7*).

We applied molecular tools to investigate the presence of zoonotic hookworms in 220 dogs from 6 parishes in Grenada. The Institutional Animal Care and Use Committee of the St. George’s University, Grenada, approved the study (approval no.: IACUC-21001-R).

## The Study

Fecal samples collected from the rectal ampulla of 220 dogs were preserved in 70% ethanol for molecular analysis. We isolated genomic DNA from the samples (≈200 mg each) by using the QIAmp PowerFecal Pro DNA Kit (QIAGEN, https://www.qiagen.com), eluted into 50 μL (instead of 100 μL) of solution C6, and then stored the samples at –20°C. To differentiate the hookworm species, we subjected individual DNA samples to M-qPCR (*3*) and analyzed and displayed the data by using GraphPad Prism version 8.0 (GraphPad, https://www.graphpad.com). Samples positive for *A. ceylanicum* hookworm DNA by quantitative PCR underwent conventional PCR and Sanger sequencing targeting a partial region of the cytochrome oxidase subunit 1 (*cox*1) gene as previously described (*8*). To analyze sequences, we used Geneious prime 2021, version 2.2 (https://www.geneious.com).

Overall, of the 211 samples for which DNA isolation was successful, 40.8% (95% CI 34.1%–47.3%) were positive for >1 hookworm species: 27.5% (95% CI 21.4%–33.5%) *A. caninum*, 4.7% (95% CI 1.87%–7.61%) *A. ceylanicum*, and 8.5% (95% CI 4.76%–12.3%) both parasites. Neither *A. braziliense* nor *Uncinaria stenocephala* DNA was detected (Figure). BLAST (https://blast.ncbi.nlm.nih.gov) analyses of *cox*1 sequences showed 100% nt identity with sequences of *A. ceylanicum* hookworms isolated from humans (GenBank accession nos. MK7928230-35) and dogs (GenBank accession no. LC533320). A representative *cox*1 sequence of *A. ceylanicum* hookworms found in this study was deposited in GenBank under accession no. OP077312.

**Figure:**
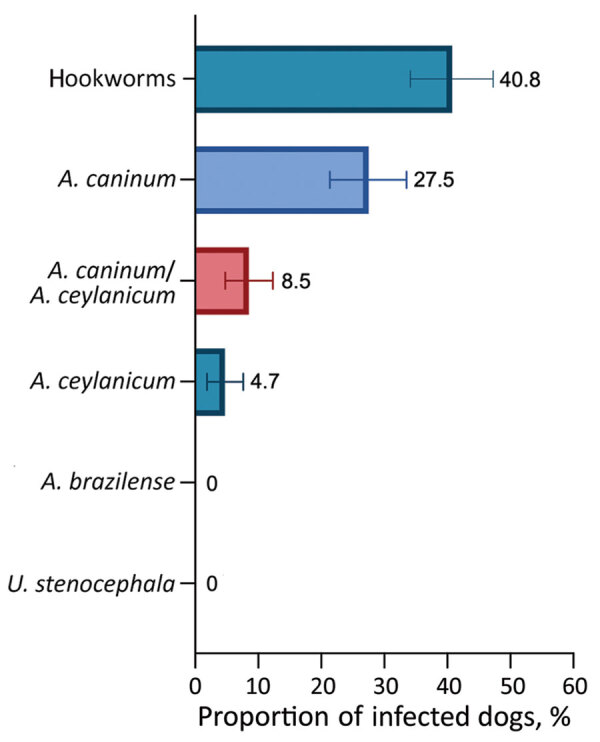
Proportion of dogs infected with zoonotic hookworms in Grenada, 2021, determined by using multiplex quantitative real-time PCR. Error bars indicate 95% CI. *A*., *Ancylostoma*; *U*., *Uncinaria*.

## Conclusions

Molecular analysis identified *A. ceylanicum* hookworms in dogs in the Americas. In the past decade, unexpectedly high prevalence and distribution of *A. ceylanicum* hookworms in dogs, wildlife, and humans has been reported mainly in the Asia–Pacific region but also in Africa (*1*,*2*,*9*). *A. ceylanicum* was erroneously considered synonymous with *A. braziliense,* until the 2 hookworm species were differentiated on the basis of configuration of the lateral bursal rays of the male (*1*). Hence, the “emergence” of *A. ceylanicum* hookworms may be associated with the increased use of molecular tools to specifically identify and differentiate these parasites in areas where multiple species of hookworm co-exist (*1*,*2*).

In the Americas, *A. ceylanicum* hookworms have not been considered etiologic agents of human infection, despite isolation of adult worms in 4 of 64 human cadavers in the Amazonas, Brazil, in the early 1920s and, more recently, reported detection of this parasite in travelers returning from countries in Latin America (e.g., Colombia and French Guiana) to France (*1*,*5*,*10*). Of note, the geographic distribution of *A. ceylanicum* hookworms in dogs strongly mirrors that in humans in the Asia–Pacific region (*2*). To date, only a few coproscopic studies of hookworms in dogs in Grenada have been undertaken (*11*), but absence of molecular investigation raises questions as to the specific identity of the hookworms on the island.

In humans, other species of hookworms of canids can cause hookworm-related cutaneous larva migrans. *A. braziliense* hookworms cause severe serpiginous “creeping eruptions,” and *A. caninum* hookworms cause peripheral eosinophilia and aphthous ileitis (*1*). Although recent cases of long-lasting hookworm-related cutaneous larva migrans have been reported in the Caribbean, including Grenada (*11*,*12*), Martinique, Jamaica, and Brazil (*1*), we did not detect *A. braziliense* hookworm infection in our canine cohort. Nonetheless, cases of hookworm-related cutaneous larva migrans in some countries in Latin America have been attributed to hookworms of canids, despite lack of molecular evidence. For instance, 4 cases were detected in 1983 during a hookworm outbreak among US military soldiers returning from Grenada, but the etiology of these infections was not established (*11*). Furthermore, although some circumstantial evidence indicates that *A. caninum* hookworms cause patent infections in humans (*9*,*13*), molecular evidence is lacking, which is particularly pertinent given the close genetic relatedness of *A. caninum* and *A. duodenale* hookworms (*1*,*13*). Thus, use of molecular methods to specifically detect, identify, and differentiate is crucial for surveillance of hookworm infections/disease in dogs and humans and for monitoring the success of control campaigns in hookworm-endemic areas (*14*).

Molecular detection of *A. ceylanicum* and *A. caninum* and the absence of *A. braziliense* hookworms in dogs in Grenada demonstrates the value of using molecular techniques to accurately identify hookworm species in dogs and humans living in the Americas. The climate in Grenada is similar to that in other countries where prevalence of zoonotic hookworms is high (*1*,*6*,*15*). According to the World Bank in Grenada, 34.3% of its population still live in households considered multidimensionally poor, where solid waste management and public sewer systems remain inadequate (*6*). Those factors, together with lack of appropriate veterinary care for dogs and closeness of human–dog interactions, favor transmission of zoonotic pathogens to humans. Given the significance of *A. ceylanicum* hookworms as agents of human hookworm infection in the Asia–Pacific region (*1*,*2*), we advocate for active surveillance of this zoonotic parasite in dogs and humans living in tropical and subtropical countries in the Americas by using reliable molecular diagnostic tools targeting appropriate genetic markers.
